# From prehospital care to the emergency department: current status and future directions of rapid EEG and artificial intelligence in the early recognition of status epilepticus

**DOI:** 10.3389/fneur.2026.1806106

**Published:** 2026-04-13

**Authors:** Aidi Luo, Zheng Liu, Wulan Ao, Jiaxin Tang, Zucai Xu, Hao Huang

**Affiliations:** 1 Department of Neurology, The Affiliated Hospital of Zunyi Medical University, Zunyi, Guizhou, China; 2 The Second People’s Hospital of Guiyang, Guiyang, Guizhou, China; 3 Key Laboratory of Brain Function and Brain Disease Prevention and Treatment of Guizhou Province, Zunyi, Guizhou, China

**Keywords:** artificial intelligence, emergency department, nonconvulsive status epilepticus, point-of-care EEG, prehospital care, rapid EEG, status epilepticus

## Abstract

Status epilepticus (SE) represents a time-sensitive, life-threatening neurological emergency. Among its major subtypes, nonconvulsive status epilepticus (NCSE) poses particular diagnostic challenges due to subtle and highly heterogeneous clinical manifestations, frequently resulting in treatment delays and increased risk of adverse outcomes. While conventional electroencephalography (EEG) remains the diagnostic gold standard, timely access to interpretable EEG recordings in emergency department and prehospital settings is often constrained by limited availability of equipment, trained technologists, and neurophysiology expertise. Rapid EEG systems—typically using reduced electrode montages and streamlined application—have emerged to shorten the interval between clinical suspicion and acquisition of actionable EEG data, including point-of-care EEG (POC-EEG). Concurrently, artificial intelligence (AI) has been integrated into EEG analysis platforms to automate detection of epileptiform discharges and quantify seizure burden, thereby mitigating resource constraints associated with real-time interpretation. This narrative review synthesizes technological advances, clinical evidence, and key challenges related to rapid EEG and AI for early recognition of SE/NCSE. Importantly, rapid EEG—whether used alone or with AI-assisted analysis—is designed to augment and support clinical decision-making rather than supplant human expertise. Despite its considerable potential, broad clinical implementation faces challenges related to technical reliability, clinical validation, and ethical concerns.

## 1 Introduction

Status epilepticus (SE) represents the most severe expression of epileptic seizures and constitutes a time-critical neurological emergency. The International League Against Epilepsy (ILAE) definition emphasizes two critical temporal thresholds—t1 (indicating treatment initiation) and t2 (signaling increased risk of long-term consequences)—underscoring the fundamental principle that “time is brain” (1, 2). Delayed treatment of SE is associated with increased mortality and neurological morbidity (2, 3). Nonconvulsive status epilepticus (NCSE), a critical SE subtype, may manifest with impaired consciousness, delirium, behavioral disturbances, or unexplained coma, rendering it both common and diagnostically challenging in emergency settings (4, 5). Reported incidence ranges from 32 to 85 cases per 100,000 population (6, 7). Among emergency department patients with unexplained altered mental status undergoing EEG evaluation, NCSE is detected in 8 to 48% of cases, though this wide range reflects variability in patient selection, inclusion criteria, recording duration, and interpretation standards (8). Importantly, a substantial proportion of patients treated for convulsive status epilepticus (CSE) continue to have electrographic seizures after apparent clinical cessation, including NCSE, underscoring the risk of “silent” ongoing seizure activity without timely EEG monitoring (9). Without timely EEG monitoring, these occult seizures may remain undetected, resulting in missed opportunities for optimal therapeutic intervention.

**Figure 1:**
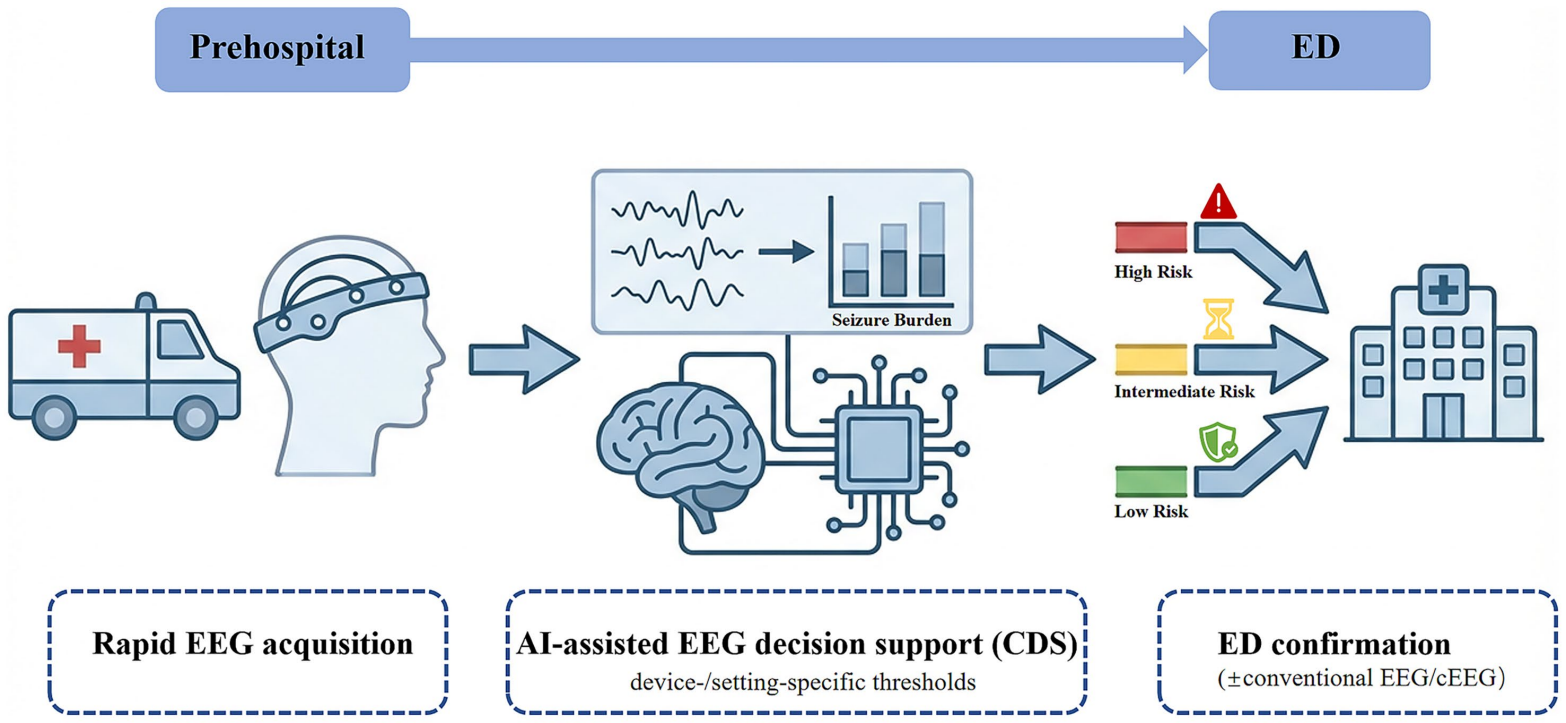
Conceptual framework of the prehospital-to-emergency department (ED) continuum integrating rapid EEG and AI. Rapid EEG is deployed in the prehospital setting (e.g., ambulance) for patients with suspected status epilepticus (SE), nonconvulsive SE (NCSE), or unexplained altered mental status. AI-assisted clinical decision support (CDS) stratifies risk levels (low/intermediate/high) to facilitate pre-arrival notification, triage, and resource mobilization. Upon ED arrival, management is guided by clinical assessment integrated with conventional EEG and/or continuous EEG (cEEG) as indicated. Risk thresholds and action protocols require calibration to specific device performance and local workflows; AI outputs function as decision support and must be interpreted within the clinical context, with expert review mandated for uncertain or high-risk cases.

Conventional EEG (e.g., the standard 10–20 system) requires specialized personnel for electrode placement and interpretation, often resulting in delays of several hours (10). In emergency and prehospital settings, immediate EEG availability is typically limited due to equipment scarcity and lack of neurophysiology expertise, compelling clinicians to make critical decisions without EEG confirmation (11). This diagnostic uncertainty leads to both overtreatment (empirical antiseizure therapy in patients without active seizures) and undertreatment (undetected persistent NCSE) (5, 8, 12). International guidelines therefore recommend urgent EEG monitoring in patients with persistent altered consciousness following convulsive seizures (13). These clinical gaps have driven the development of rapid EEG systems designed for deployment by non-specialists in acute-care environments. Rapid EEG typically employs simplified montages and headset- or patch-based hardware to reduce setup time, while AI-based analysis can provide near–real-time detection of epileptiform activity and quantification of seizure burden to support early triage and clinical decision support (CDS) (8, 14). Importantly, rapid EEG and AI are intended to augment—not replace—clinical expertise. This review summarizes current technologies, clinical evidence, and key implementation challenges of rapid EEG and AI-assisted EEG analysis across the prehospital-to-emergency care continuum, and presents a representative workflow illustrating this integration (Figure 1).

Literature search strategy and scope. This article was prepared as a narrative review. To improve transparency, we conducted a targeted literature search in PubMed, Embase, Web of Science and Google Scholar for publications from January 2015 through December 2025 using combinations of the terms “status epilepticus,” “nonconvulsive status epilepticus,” “rapid EEG,” “point-of-care EEG,” “artificial intelligence,” “machine learning,” “prehospital,” “ambulance,” “emergency department,” and “critical care.” We additionally screened the reference lists of key guidelines, systematic reviews, meta-analyses, multicenter clinical studies, and technical validation reports to identify further relevant publications. We prioritized human studies and clinically informative publications relevant to early recognition, diagnostic performance, workflow implementation, and treatment decision-making. This review was not designed as a PRISMA-based systematic review, and no formal risk-of-bias scoring or quantitative meta-analysis was performed.

## 2 Why SE is missed: clinical heterogeneity and barriers to timely EEG

### 2.1 Clinical heterogeneity and diagnostic challenges

The clinical spectrum of SE is broad, ranging from overt generalized convulsions to subtle manifestations such as facial twitching or minor alterations in behavior or consciousness (1, 2). NCSE may be obscured by underlying conditions including stroke, trauma, hypoxia, or infection, or may present with nonspecific features such as coma or delirium (2, 4, 15). In time-pressured emergency settings, these presentations are easily attributed to alternative etiologies, contributing to underrecognition and diagnostic delay (5, 8). While neuroimaging (CT/MRI) can elucidate structural causes of altered mental status, it cannot confirm ongoing epileptic activity, rendering EEG indispensable (16). The central challenge is that, in the absence of overt convulsions, clinicians often cannot confidently distinguish NCSE from other causes of encephalopathy without electrographic evidence, resulting in delayed or inappropriate treatment decisions.

### 2.2 Limited accessibility of conventional EEG in emergency and prehospital care

Although continuous EEG (cEEG) represents the gold standard for diagnosing NCSE, quantifying seizure activity, and guiding therapy, its deployment in emergency departments and prehospital settings faces substantial practical barriers. These include prolonged initiation times (often hours), cumbersome equipment, operational complexity, and dependence on expert interpretation—limitations particularly pronounced in community hospitals and resource-constrained regions (17–19). Even in well-resourced healthcare systems, delays from emergency presentation to EEG initiation remain commonplace, forcing clinicians to rely on empirical treatment or transfer decisions in the absence of definitive diagnostic evidence (20). Benzodiazepines are first-line therapy for SE. Early administration (e.g., within 5–10 min of seizure onset) increases the likelihood of seizure termination, whereas delayed dosing reduces efficacy and increases the risk of progression to refractory SE (13, 21–23). Recommended first-line benzodiazepines include diazepam, lorazepam, and midazolam, which can be administered via multiple routes (intravenous, intramuscular, intranasal, or buccal) to achieve rapid therapeutic effect (24–26). NCSE presents a distinct clinical dilemma: determining when to initiate immediate treatment for suspected impaired consciousness, when to prioritize cEEG confirmation, and when to avoid excessive empirical sedation or intubation (4, 27). In the absence of EEG guidance, clinicians must balance the risks of delayed therapy and secondary neurological injury against inappropriate antiseizure or sedative exposure in non-epileptic conditions, with attendant adverse effects and resource utilization. Given the time-critical nature of SE, there exists a clear imperative for rapid, reliable diagnostic tools in prehospital and emergency environments. Bedside rapid EEG, coupled with remote expert review and/or AI-enabled decision support, may provide timely evidence of ongoing epileptiform activity and facilitate a tiered “screen-first, confirm-later” diagnostic strategy (28).

## 3 Rapid EEG devices and AI-based seizure detection: what does the evidence show?

### 3.1 Advances in rapid EEG technology

In this review, we use rapid EEG as an umbrella term for EEG systems designed for expedited deployment in acute-care environments, and use point-of-care EEG (POC-EEG) to refer specifically to bedside, clinician-deployed rapid EEG platforms used to support immediate clinical decision-making; when applicable, the terms overlap because many POC-EEG devices are rapid EEG systems. Several rapid EEG systems have been developed to enable rapid deployment through simplified electrode configurations and predefined placements. For instance, the Ceribell system uses a 10-electrode headband and can typically be applied in ~5 min; when paired with its AI-based Clarity algorithm, it provides real-time alerts, seizure-burden trending, and remote review capabilities (12, 28, 29). The Zeto system adopts a wearable array that more closely approximates the spatial coverage of the standard 10–20 layout and reduces external wiring through a dry-electrode design and streamlined setup protocols to improve bedside usability (30). Nevertheless, recording quality and automated detection performance require continued evaluation across heterogeneous acute-care environments. Research indicates that with minimal training, emergency nurses or physicians can complete device setup and initiate recordings significantly faster than with conventional EEG (12, 31, 32). A narrative review including 69 studies further concluded that POC-EEG can accelerate recognition of suspected NCSE and facilitate earlier diagnostic clarification, particularly in time-critical or resource-limited settings (33). A major practical advantage of these devices is their compatibility with telemedicine workflows, enabling EEG data transmission to remote neurologists and/or AI platforms when on-site neurophysiology coverage is unavailable. However, the trade-off of reduced electrode montages is diminished spatial resolution and decreased sensitivity for detecting focal or region-specific seizure activity (such as parietal, central, or certain temporal lobe discharges) (28, 34). A meta-analysis by Lin et al. found that simplified EEG using 8–10 electrodes achieved a pooled sensitivity of approximately 75% for seizure detection, suggesting inferior performance compared to standard full-montage EEG (35). Consequently, the clinical role of rapid EEG is better suited as a “rapid screening and early triage tool” for initial assessment and disposition decisions in high-risk patients, rather than as a complete replacement for cEEG used for precise localization and prolonged monitoring. When rapid EEG identifies seizures or highly epileptiform patterns, confirmatory testing and definitive treatment planning should follow; conversely, the absence of epileptiform activity may help deprioritize NCSE and avoid unnecessary escalation while alternative etiologies are evaluated (31, 36–38). Beyond SE/NCSE, rapid EEG applications are expanding to other acute neurological conditions, including traumatic brain injury, stroke, and delirium, for diagnostic and prognostic purposes (11, 39). Throughout this review, we generally distinguish between rapid EEG—referring to the hardware platform and streamlined acquisition process—and AI-assisted EEG analysis, which represents a separate analytical layer that may or may not be integrated with these devices. Where relevant, we specify whether a given study employed AI-based tools, clinical risk scores, EEG sonification, or rapid EEG hardware alone.

### 3.2 AI in EEG analysis and diagnostic performance

Artificial intelligence (AI), encompassing machine learning (ML) and deep learning (DL), is increasingly being integrated into EEG workflows and is reshaping neurological diagnostics (14, 40). Various algorithms have been applied to EEG data, including classical machine learning models such as support vector machines (SVM), as well as modern deep learning architectures including convolutional neural networks (CNN), recurrent neural networks (RNN), and long short-term memory (LSTM) networks (14, 40, 41). These methods have been applied to artifact suppression, automated feature learning, pattern classification, event detection, and real-time alerting based on EEG signals (41–43). AI-EEG applications can generally be categorized as either supportive AI, which assists clinicians in detecting and diagnosing epileptic seizures (essentially decision support), or predictive AI, which aims to forecast seizure occurrence or outcomes before clinical manifestation (44). Current clinical translation has largely focused on the former. For suspected NCSE, models trained on labeled EEG patterns can detect epileptiform discharges (e.g., spikes and sharp waves) and ictal rhythmic activity. These algorithms can also calculate quantitative metrics, such as the proportion of time during which epileptic activity occurs (seizure burden), providing an objective measure of seizure frequency within the brain’s electrical activity. Such outputs may be reported as a continuous percentage or as threshold-based categories, enabling rapid risk stratification and clinical decision support.

Evidence from large conventional EEG datasets suggests that AI can approach expert-level performance for detecting epileptiform abnormalities. In a multicenter study of >30,000 EEG recordings, a deep CNN-based system (SCORE-AI) classified EEGs as normal or abnormal and differentiated epileptiform patterns (generalized and focal discharges), with performance comparable to that of expert clinicians (41). The system achieved AUCs of approximately ~0.89–0.96 for detecting epileptiform activity—comparable to consensus interpretation by expert readers (41). Specificity (~90%) was slightly higher than that of human experts, while sensitivity and overall accuracy were similar (41). In the more specific setting of nonconvulsive seizures/NCSE, a recent systematic review and meta-analysis of 12 studies reported pooled sensitivity of ~95% and specificity of ~83%, using expert EEG interpretation as the standard (28). While such sensitivity is attractive for screening, false positives remain a concern when the pretest probability of NCSE is low-to-moderate. The meta-analysis projected that in high-risk emergency patients, with a typical pre-test probability of 40%, this AI system would produce one false positive for every seven patients without seizures, supporting the use of AI primarily for screening and prioritization rather than as a stand-alone basis for treatment escalation (28).

These performance estimates warrant careful interpretation. SCORE-AI was trained and validated on conventional full-montage EEG, controlled recording conditions, curated datasets— and the extent to which such results transfer to rapid EEG systems is unclear (41). Rapid devices use fewer electrodes (typically 8–10), cover less of the scalp, and are far more exposed to motion artifact, muscle contamination, and electromagnetic noise than their conventional counterparts (31, 35, 43). The performance gap is not trivial. Lin et al.’s meta-analysis of reduced-montage EEG reported a pooled seizure detection sensitivity of roughly 75%, well below full-montage benchmarks, and a more recent emergency-focused meta-analysis found POC-EEG sensitivity as low as 69% (95% CI: 55–80%), though specificity remained high at 99% (95% CI: 95–100%) (35) Fewer electrodes mean less spatial resolution, and the artifact burden inherent to acute-care environments—ambulance vibration, patient movement, poor electrode-skin contact—compounds the problem in ways that laboratory recordings do not capture. Limited model interpretability and data quality constraints add further barriers to clinical translation. There is also the issue of domain shift: algorithms developed on clean, annotated datasets from academic centers may not perform comparably when applied to recordings from community emergency departments, where equipment varies, patient populations differ, and concurrent video for artifact adjudication is typically unavailable (28, 31, 42, 43). For these reasons, diagnostic metrics derived from conventional full-montage EEG should not be assumed to hold for rapid EEG deployed under field conditions; external validation across devices, institutions, and patient populations remains a necessary step before clinical adoption (31, 41, 43).

### 3.3 AI’S seizure burden threshold and alert performance

A key operational question for AI-enabled rapid EEG is how seizure alerts are triggered. Many platforms issue alarms once the estimated seizure burden exceeds a preset threshold, and changing this threshold entails an inherent trade-off between sensitivity and specificity. Real-world evaluations illustrate this balance. In a retrospective analysis of 353 rapid EEG recordings, using a seizure-burden threshold of 10%, the Clarity algorithm achieved 88% sensitivity and 60% specificity for SE detection (29). Lower thresholds may facilitate earlier detection but increase false positives and the risk of alarm fatigue. Similarly, in the AccuRASE study (*n* = 235), an automated seizure-burden estimator achieved 86% sensitivity and 79% specificity for identifying patients with >10% seizure burden within the first 2 h of recording (45). Collectively, these data support tailoring thresholds to the clinical objective and care setting. Higher thresholds (e.g., >90%) may be considered to prioritize specificity and improve the positive predictive value of alerts, thereby helping prioritize patients for expedited expert review and escalation to cEEG when electrographic seizures are strongly suspected. From a clinical management perspective, seizure-burden thresholds should be interpreted as triage thresholds rather than stand-alone treatment triggers. Lower thresholds (e.g., >10%) are best suited to screening because they favor sensitivity and negative predictive value; In the absence of epileptiform activity, clinicians may reasonably deprioritize NCSE and pursue alternative diagnostic workup, potentially avoiding unnecessary antiseizure medication exposure and its attendant risks (respiratory depression, hypotension, unnecessary ICU admission). However, reduced-montage sensitivity limitations should be acknowledged, and repeat or prolonged EEG should be considered if clinical suspicion remains high (28, 31, 45). Medium thresholds (e.g., 10–90%) may have a relatively high false positive rate; these findings should prompt urgent neurology consultation and initiation of cEEG rather than immediate treatment escalation. First-line benzodiazepine therapy may be appropriate based on clinical judgment, but escalation to anesthetic coma should await EEG confirmation. By contrast, higher thresholds (e.g., >90%) prioritize specificity and may support immediate neurologist notification, expedited confirmation, and parallel preparation for escalation when electrographic seizures are strongly suspected and the clinical context is concordant (28, 45). Importantly, in patients with overt convulsive SE, treatment should follow time-sensitive guideline-based management without waiting for AI outputs, whereas in suspected NCSE, AI alerts must be integrated with bedside examination, clinical trajectory, signal quality, alternative diagnoses, and confirmatory EEG review whenever feasible (13, 21–23, 31).

### 3.4 Impact of rapid EEG—with and without AI—on clinical decision-making and patient outcomes

Beyond diagnostic accuracy, the clinical value of rapid EEG lies in accelerating workflows and informing management decisions in time-critical settings. It is important to note that this evidence base spans several technological approaches—including AI-based automated analysis, EEG sonification, and clinical risk scores. A meta-analysis of studies employing AI-enabled EEG platforms reported that, in emergency contexts, the median time to first EEG was approximately 242 min with conventional workflows, whereas rapid EEG was typically initiated within ~1 h of presentation, this difference is mainly attributed to the rapid deployment of hardware equipment rather than the AI analytical layer (28). Rapid EEG may also reduce unnecessary exposure to antiseizure medications (ASMs). Across four prospective cohort studies (n = 298), POC-EEG was associated with a lower risk of unnecessary ASMs administration (risk difference = −0.26; 95% CI: −0.50 to −0.02), although the relative contributions of earlier EEG availability and AI-assisted interpretation to this effect cannot be disentangled from the pooled data (28). Avoiding empirical escalation in patients ultimately found not to be seizing may reduce medication-related adverse events (e.g., respiratory depression from sedatives/anesthetics) and prevent potentially avoidable intubation, sedation, and ICU utilization (42, 46). However, heterogeneity was substantial (*I*^2^ ~ 82%), suggesting that effect sizes likely depend on patient selection and implementation pathways.

Several studies further demonstrate that rapid EEG often leads to changes in clinical management, though the technology behind these workflow gains varies. In the ICU, Hobbs et al. used rapid EEG with EEG sonification (the Brain Stethoscope function, not AI-based automated detection) and reported treatment adjustments in ~40% of cases and a reduction in inappropriate treatment in ~25% (46). Similarly, the DECIDE multicenter prospective study employed rapid EEG with sonification—without AI tools—and found that it altered planned management in ~20% of ICU/ED patients while increasing diagnostic confidence among treating teams (47). In an ED pilot study (*n* = 38) of suspected NCSE, clinicians reported that rapid EEG with sonification (without AI-based automated analysis) influenced management in 53% of cases, most commonly by helping rule out SE and thereby avoiding escalation to sedatives or intravenous ASMs and expediting downstream disposition (e.g., safe transition from resuscitation to observation) (20). It is worth noting, however, that faster workflows do not automatically translate into better patient outcomes. The studies cited above primarily measure process endpoints—time to EEG, diagnostic confidence, changes in management, transfers avoided—not mortality, functional recovery, or long-term seizure control (20, 28, 46–48). The outcome literature that does exist is observational, with all the usual vulnerabilities to confounding by illness severity, case selection, and local practice patterns. The SAFER trial subanalysis is a case in point: Desai et al. evaluated early rapid EEG findings using the 2HELPS2B clinical risk score—again without AI-based seizure detection—and propensity score matching showed shorter ICU stays (3.9 vs. 8.0 days) and better neurological status at discharge (mRS < 4) in the POC-EEG group, but this was a retrospective, nonrandomized comparison, and mortality did not differ between groups (48). These results are best read as hypothesis-generating. No completed RCT has yet shown that rapid EEG or AI-enabled monitoring improves mortality, neurological function, or post-discharge seizure control over standard care. The Tele-cRCT study, tele-continuous EEG detected more nonconvulsive seizures/status epilepticus than tele-routine EEG, but mortality and functional outcomes did not differ significantly between groups, underscoring that increased seizure detection does not necessarily translate into better clinical outcomes (49). Closing this evidence gap will require prospective multicenter trials powered for hard clinical endpoints: mortality, functional outcome, seizure cessation, ASM and sedative exposure, intubation rates, length of stay, interfacility transfer, and cost-effectiveness (28, 48).

## 4 Rapid EEG along the care continuum: prehospital triage and emergency department workflows

**Figure 2:**
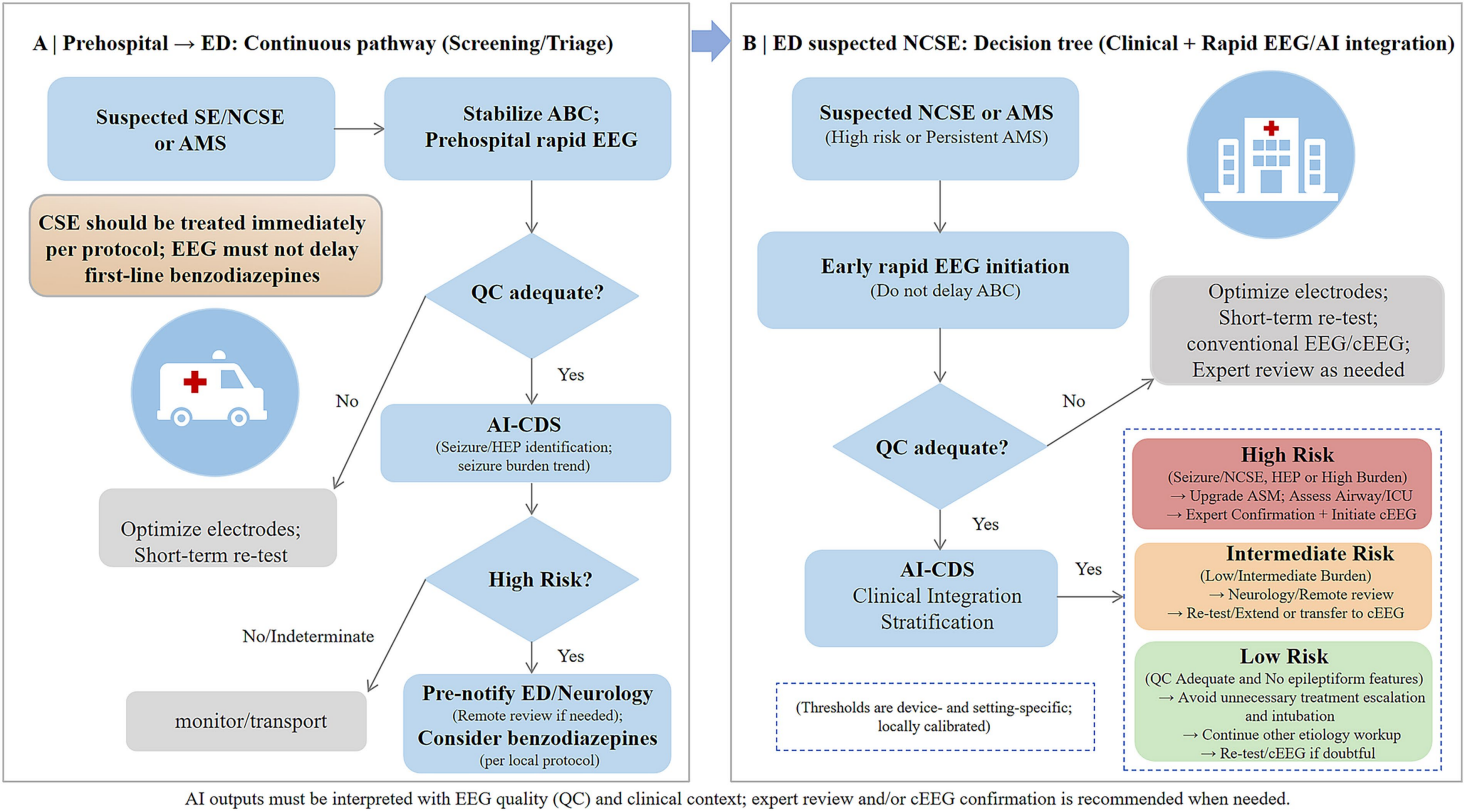
Tiered workflow and decision tree for integrating rapid electroencephalography (EEG) with artificial intelligence (AI) from prehospital screening to emergency department (ED) confirmation. This figure is intended as a conceptual workflow rather than a study-by-study evidence map. **(A)** Prehospital pathway: In cases of suspected status epilepticus (SE), nonconvulsive status epilepticus (NCSE), or unexplained altered mental status (AMS), rapid EEG is initiated after airway–breathing–circulation (ABC) stabilization. If quality control (QC) is inadequate, electrodes are adjusted and recording repeated. Interpretable AI-based clinical decision support (CDS) detects electrographic seizures or highly epileptiform patterns (HEP); high-risk outputs prompt pre-notification without delaying benzodiazepines. **(B)** ED decision tree: Upon ED arrival, rapid EEG is started promptly. EEG/AI outputs are integrated with clinical data to stratify patients into high-, intermediate-, or low-risk categories, guiding escalation, monitoring, and disposition decisions as shown. Risk thresholds and action protocols require calibration to specific device performance and local workflows; AI outputs function as decision support and must be interpreted within the clinical context.

Drawing on current evidence and acute-care needs, we propose a tiered workflow and decision tree spanning prehospital screening and ED confirmation (Figure 2). This framework highlights key junctures for signal quality control (QC), AI-based risk stratification, and timely expert review and/or escalation to cEEG.

### 4.1 Rapid EEG in the ambulance: feasibility, signal quality, and triage potential

The prehospital setting offers a unique opportunity to obtain electrophysiological evidence of ongoing seizure activity before hospital arrival, enabling early ED pre-notification, triage, and resource mobilization. In the PHIRE mixed-methods feasibility study, emergency medical services personnel applied POC-EEG in the field with a median setup time of 2.5 min, and 94% of recordings were interpretable (32). While encouraging, the prehospital evidence base remains limited: sample sizes are small, and randomized comparisons and rigorous health-economic evaluations are largely lacking. In principle, prehospital rapid EEG—particularly when coupled with real-time AI decision support—could shorten the interval from symptom onset to actionable EEG evidence, supporting earlier treatment decisions and more informed transport planning; however, direct benefits on neurological outcomes have not yet been demonstrated (32, 50). AI-assisted real-time analysis may provide EMS teams with actionable prompts during care. A high estimated seizure burden could reinforce the decision to initiate or continue benzodiazepines, whereas an absence of epileptiform activity may prompt consideration of alternative etiologies (e.g., stroke, hypoglycemia) and help avoid unnecessary sedation (22, 23). AI approaches have also shown potential for distinguishing epileptic from non-epileptic events (including psychogenic seizures), although evidence specific to the prehospital environment remains sparse (51, 52). Although prehospital studies focused on NCSE are still scarce, POC-EEG is also being investigated for stroke triage (e.g., identifying large-vessel occlusion and common mimics), including prehospital and transport studies, suggesting broader applicability across neurologic emergencies (39). Operational workflows may include rapid EEG setup in ambulances, real-time transmission to an AI/cloud platform, automated analysis, and immediate feedback to the treating team, thereby extending neuro-monitoring into the prehospital phase (50).

### 4.2 Rapid EEG in the emergency department: guiding triage, treatment, and disposition

Upon arrival at the emergency department, patients with suspected SE or NCSE require prompt evaluation and management. In cases of convulsive status epilepticus (CSE), well-established clinical guidelines and overt motor manifestations allow emergency clinicians to initiate aggressive treatment early, often without the need for immediate EEG confirmation (53, 54). In contrast, NCSE presents a distinct clinical dilemma: in the absence of EEG evidence, clinicians may administer benzodiazepines empirically but frequently hesitate to escalate therapy to anesthetic coma or high-dose antiseizure treatment without confirmation of ongoing ictal activity (4, 55, 56). This is where rapid EEG can function as a practical “decision accelerator” (57, 58). Rapid/point-of-care EEG adds value not only by shortening time to actionable neurophysiologic data, but also by enabling early seizure/ictal-activity risk stratification—whether through clinical risk scores such as 2HELPS2B (37, 38) or AI-based seizure-burden quantification— and pathway redesign (29, 36–38). Early bedside EEG can categorize patients by electrographic findings and/or EEG-based risk tools (e.g., 2HELPS2B) into low-, intermediate-, or high-risk groups for electrographic seizures and possible NCSE, guiding prioritization of expert review and the need/duration of cEEG (31, 36, 38). High-risk patterns (e.g., electrographic seizures or clearly highly epileptiform/high-risk features on screening EEG) should prompt expedited expert review and consideration of escalation of antiseizure therapy, along with initiation of cEEG and higher-acuity monitoring when clinically indicated (31, 36–38). Intermediate or equivocal findings may warrant early neurology consultation and repeat or prolonged EEG to clarify seizure risk and inform treatment and monitoring decisions (36–38). Conversely, a low-risk rapid EEG (e.g., no epileptiform features, with normal background or nonspecific diffuse slowing) can support a more conservative approach, potentially reducing unnecessary antiseizure escalation while alternative etiologies are evaluated; however, limitations of reduced-montage rapid EEG and automated detection underscore the importance of clinical context and timely expert confirmation when results are discordant or management-changing (31, 36, 37, 41).

Hospital-based studies provide proof-of-concept for these workflow benefits. A nurse-led quality-improvement initiative in a community hospital reduced median time to EEG initiation from 562 to 74 min after implementing bedside rapid EEG, and electrographic seizures were identified in 23% (27/115) of evaluated patients (59). Rapid EEG may also reduce interfacility transfers prompted solely by concern for NCSE. In a two-year implementation program (*n* = 189), seizures were detected in 13.2% of recordings, and only 1.1% of patients had seizures identified only on subsequent conventional EEG (60). In a multicenter retrospective comparative-effectiveness study, rapid EEG showed similar performance to conventional EEG for early seizure-risk stratification using the 2HELPS2B score (AUC ~ 0.85) (38). This suggests that rapid EEG can serve as an early monitoring and resource allocation tool. The 2HELPS2B score, which was initially validated in conventional EEG settings, shows that the combination of rapid EEG with 2HELPS2B is methodologically sound and provides a practical approach to determining which patients need extended EEG monitoring during the transition from the emergency department to ICU (37). In an ED pilot study (*n* = 38) of suspected NCSE, clinicians reported that rapid EEG helped avoid treatment escalation or intubation in over half of cases because no seizures were detected (20). This benefit reduces unnecessary ICU admissions and sedation, while also freeing up ICU resources and reducing costs. However, the small number of NCSE events limits the study’s conclusions to feasibility and workflow benefits rather than definitive prognostic outcomes (20). While these studies highlight feasibility and workflow impact, outcome evidence remains limited and larger prospective trials are needed. In pediatric ED care, POC-EEG has likewise been reported to influence management decisions, although heterogeneity remains and further prospective evidence is required (61, 62). Table 1 summarizes the representative clinical evidence supporting the use of rapid EEG and AI throughout the entire prehospital-to-emergency process, highlighting their primary role as early screening and triage tools, rather than as substitute diagnostic methods for conventional EEG.

**Table 1:** Clinical utility of rapid EEG and AI for early recognition and triage of SE/NCSE across the prehospital–to–emergency care continuum.

Setting/phase	Study (reference)	Design (*n*)	Technology evaluated	Population/indication	Index test (reference standard)	Main findings and clinical impact
Prehospital and ED						
Prehospital (ambulance)	Guterman et al. (32)	Mixed-methods feasibility study (PHIRE); *n* = 34	Rapid EEG only	Suspected seizures/altered mental status in EMS setting	POC-EEG (Ceribell) applied and recorded by EMS; reference: neurologist review when available	Feasibility: median device setup time 2.5 min; interpretable EEG in 94% Implication: supports feasibility of an integrated prehospital–ED pathway
ED	Wright et al. (20)	Prospective pilot study; *n* = 38	Rapid EEG + sonification	Suspected NCS/NCSE	Rapid EEG (Ceribell) + Brain Stethoscope (sonification); reference: neurologist/expert interpretation	Clinical impact: management changed in 20/38 (53%); care accelerated in 8/38 (21%) Diagnostic performance: Se 100%, Sp 92% Limitation: very few outcome events, wide confidence intervals
ED	Kozak et al. (12)	Retrospective cohort; *n* = 157	Rapid EEG + AI	Suspected NCS/NCSE	POC-EEG (Ceribell) + Clarity algorithm (bedside/remote over-read); reference: expert interpretation	Workflow gain: median time to POC-EEG ~ 1.2 h; 55% after-hours Decision-making: epileptiform activity group (seizures/HEP) more often had ASMs escalation (52% vs. 25%); nonepileptiform group more often avoided escalation (27% vs. 2%)
ED	Richard et al. (31)	Single-center prospective observational; *n* = 108	Rapid EEG + AI	Altered mental status of unclear etiology (acute neurological symptoms)	Rapid EEG + AI seizure burden detection (threshold >90% seizure burden); reference: epileptologist interpretation	Prevalence: NCSE/SE confirmed in 3 cases AI performance: Se 66.7%, Sp 97.0%, accuracy 96.1% Implication: AI may overestimate seizure burden in some cases; expert confirmation remains necessary
ED	Welte et al. (92)	Retrospective cohort; *n* = 100	Rapid EEG only	Suspected seizures /or altered consciousness	Simplified wireless 8‑channel EEG (swEEG) by non-specialists; reference: conventional EEG	Feasibility: median application 7 min Validity: swEEG matched conventional EEG category in 87.3% (48/55) Implication: simplified wireless systems may serve as adjunct screening in resource-limited EDs
ED	Eberhard et al. (59)	Nurse-led QI; pre/post comparison (6 months each); rapid EEG *n* = 115 vs. conventional EEG *n* = 35	Rapid EEG only	Suspected seizures or unexplained altered consciousness	Rapid EEG (Ceribell); reference: conventional EEG workflow and interpretation	Workflow: median time to EEG initiation reduced by 7.6-fold Detection: seizures confirmed in 23% of cases System-level: increased after-hours EEG availability (~3.29-fold)
ED (pediatrics)	Simma et al. (61)	QI study; *n* = 62	Rapid EEG only	Pediatric ED: acute CNS symptoms/suspected seizures	POC-EEG (rapid 2-channel/simplified protocol); reference: standard EEG and/or clinical assessment	Clinical impact: POC-EEG influenced 60% of clinical decisions Detection: epileptiform activity in 10/62 (16%) Agreement: ~68% (37/62) concordance with standard EEG
Community/transfer avoidance (no LTM/cEEG)						
Community hospital ED	Madill et al. (74)	Retrospective observational; *n* = 74 (118 rapid EEG studies)	Rapid EEG only	Suspected NCSE/NCS	Rapid EEG (Ceribell) with remote/on-site interpretation; reference: local neurology interpretation and transfer as needed	Transfers: among 33 “planned transfer” assessments, transfer avoided in 31 (94%) Costs: estimated savings >$39,000 (≈$1,274 per patient)
Community hospital ED/ICU	Ward et al. (80)	Prospective cohort + retrospective cohort; *n* = 88	Rapid EEG only	Suspected seizures/altered mental status	POC-EEG (Ceribell); reference: subsequent cEEG and clinical follow-up	Transfers: estimated annual reduction of 10.8 transfers to tertiary centers for cEEG Economics: net gain $13,936 per avoided transfer; annual fixed system cost $119,700 Missed-seizure risk: seizures detected on later conventional EEG in 2/83 (2.4%)
ICU/in-hospital acute care						
ICU	Vespa et al. (47)	Multicenter prospective study (DECIDE); *n* = 164	Rapid EEG + sonification	Suspected seizures/NCSE	Rapid EEG (Ceribell) + Brain Stethoscope; reference: conventional EEG workflow + expert interpretation	Workflow: median time to EEG initiation 5 min vs. 239 min Diagnosis: improved bedside clinical diagnostic accuracy (Se 77.8% → 100%; Sp 63.9% → 89%) Decision-making: planned treatment changed in 33/164 (20%)
ICU	Desai et al. (48)	Sub-analysis of a prospective cohort (SAFER trial); *n* = 283	Rapid EEG + clinical risk score	Suspected seizures/NCSE	2HELPS2B score applied to POC-EEG (Ceribell); reference: conventional EEG	Outcomes: ICU LOS 3.9 d (IQR 1.9–8.8) vs. 8.0 d (IQR 3.0–16.0) Neurologic outcome: higher proportion discharged with mRS < 4; mortality not significantly different Limitation: nonrandomized design and residual confounding; randomized trials needed
ICU	Hobbs et al. (46)	Prospective observational; *n* = 35	Rapid EEG + sonification	Suspected seizures	Rapid EEG (Ceribell) + Brain Stethoscope (sonification); reference: expert interpretation	Workflow: mean EEG setup time 6 ± 3 min Clinical impact: treatment changed in ~40%; inappropriate treatment reduced in ~25% Implication: emphasizes usability/immediacy; larger outcome studies warranted
Isolation ward	LaMonte (75)	Retrospective observational; sample size NR	Rapid EEG only	Suspected seizures/encephalopathy in COVID-19 isolation	Rapid EEG (Ceribell); reference: conventional EEG	Workflow: diagnostic/interpretation time 24 min vs. 126 min; staffing requirements reduced by ~50% Implication: highlights system value under isolation and staffing constraints
AI algorithms/validation						
ED + ICU (algorithm validation)	Kamousi et al. (29)	Retrospective methodological ML development/validation; *n* = 353	Rapid EEG + AI algorithm	Critical care settings (seizure and HEP burden monitoring)	Clarity algorithm; reference: expert EEG review	Example threshold: algorithm generated an SE alert; at 10% seizure burden, Se 88% and Sp 60% Implication: suited for screening/alerting rather than definitive diagnosis
Multicenter (algorithm validation)	Sheikh et al. (45)	Retrospective observational diagnostic performance; *n* = 235	Rapid EEG + AI algorithm	Seizure burden assessment in the first 2 h of EEG	ASBE (Automated Seizure Burden Estimator); reference: blinded expert consensus (2/3 reviewers)	Accuracy: For outcome “>10% seizure burden in first 2 h”: Se 86%, Sp 79% Implication: supports early stratification and prioritization (e.g., escalation to cEEG)
Multicenter (algorithm validation)	Tveit et al. (41)	Retrospective multicenter diagnostic accuracy; *n* = 30,493	AI algorithm	IED detection in clinical EEG	SCORE-AI to assist IED identification; reference: clinical neurophysiologist interpretation	Performance: AI achieved expert-level accuracy in conventional EEG interpretation (Se 88.3%, Sp 90.0%, AUC 0.89–0.96) Implication: supports generalizability for structured EEG reading, but requires validation in noisy ED settings
Multicenter (risk stratification)	Kalkach-Aparicio et al. (38)	Retrospective multicenter comparative effectiveness; *n* = 500	Rapid EEG + clinical risk score	Inpatient/ICU: seizure risk prediction using rapid EEG	Rapid EEG (Ceribell) enabling risk scores such as 2HELPS2B; reference: conventional EEG/expert interpretation	Risk prediction: AUC ~ 0.85 Implication: supports risk stratification within the first hour to guide prolonged monitoring and/or referral
Multiple settings (ED/ICU/resource-limited)	Gomutbutra et al. (28)	Systematic review and meta-analysis; total *n* = 1,575	Rapid EEG + AI	Suspected NCS/NCSE	AI-EEG (mixed platforms) for early detection of NCS/NCSE (commercial and noncommercial); reference: expert EEG interpretation/clinical diagnosis	Diagnostic accuracy: pooled Se 95%, Sp 83%, FPR 13.9% Workflow: absolute reduction in unnecessary ASMs use by 26% (risk difference [RD] = −0.26, 95% CI − 0.50 to −0.02). time to EEG acquisition in resource-limited environments reduced from 4.5 h (IQR 3.2–6.8) to 2.1 h (IQR 1.5–3.4) Implication: false positives remain a concern even in high pretest-probability settings (≈1 in 7)

AI, artificial intelligence; ASMs, antiseizure medications; AUC, area under the curve; cEEG, continuous EEG; ED, emergency department; EMS, emergency medical services; FPR, false positive rate; HEP, highly epileptiform patterns; ICU, intensive care unit; IED, interictal epileptiform discharge; IQR, interquartile range; LOS, length of stay; LTM, long-term EEG monitoring; NCSE, nonconvulsive status epilepticus; NCS, nonconvulsive seizures; NR, not reported; POC-EEG, point-of-care EEG; SE, status epilepticus; Se, sensitivity; Sp, specificity; QI, quality improvement; RR, risk ratio.

## 5 Current status and challenges

### 5.1 Data quality and standardization

The performance of AI-enabled EEG analysis is intrinsically dependent on data quality. Evidence from out-of-hospital and multimodal monitoring studies indicates that, under real-world conditions, the proportion of EEG segments suitable for automated analysis may be substantially reduced by noise and artifacts (63). Evidence from out-of-hospital monitoring confirms this concern, showing that a substantial proportion of prehospital EEG segments were unsuitable for automated analysis—a finding that suggests validation-study accuracy may overestimate what these systems actually deliver when deployed in ambulances or busy emergency departments (63). In prehospital and acute-care environments, electromyographic activity, eye movements, patient motion, and electromagnetic interference are more prevalent and can significantly compromise signal integrity, model stability, and reproducibility (14, 31, 64). Variability in electrode number, configuration, and spatial coverage across POC-EEG systems further affects signal representation and diagnostic performance. Reduced montages inevitably trade spatial resolution for speed and accessibility, increasing the risk of missing focal or region-specific epileptiform activity. Recent systematic reviews suggest that although reduced-montage EEG is feasible in emergency and ICU settings, sensitivity remains imperfect, and performance varies with electrode layout and clinical context (35). These limitations underscore the need for standardized acquisition protocols, explicit quality-control (QC) criteria, and clearly defined minimum requirements for interpretable recordings (43). In addition, the lack of harmonized annotation standards and shared, high-quality datasets across institutions increases the risk of overfitting and limits external generalizability of AI models (31). To ensure robust clinical performance, multicenter and cross-regional datasets are required for training, external validation, calibration, and post-deployment performance monitoring.

### 5.2 Interpretability and generalizability of AI models

Most deep learning models continue to function as “black boxes,” making it difficult for clinicians to understand the basis of alarm triggers or seizure burden estimates, which affects trust and adoption (42, 44). Even when existing systems perform well in specific cohorts, their performance may be unstable across different populations, sedation protocols, and device parameters. This concern is particularly relevant when AI models developed and validated on conventional routine EEG are considered for use in acute-care reduced-montage settings (65). External validation of SCORE-AI showed that performance remained robust across independent datasets recorded under routine full-montage conditions, but this does not establish generalizability to reduced-montage emergency recordings (65). A recent review noted, models optimized on curated, noise-free datasets lack ecological validity, and the gap between laboratory performance and real-world clinical variability remains a persistent barrier to translation. Site-specific validation, recalibration, and post-deployment surveillance are preconditions for responsible clinical use, not steps that can be deferred (66). Therefore, priority should be given to the development of explainable AI with clinical decision-oriented outputs, such as confidence scores, identification of key segments, and verifiable evidence. Furthermore, multicenter prospective validation and post-deployment surveillance are essential to ensure long-term effectiveness and reliability (31, 41, 51).

### 5.3 Ethical and privacy considerations

Rapid EEG systems—and the AI-assisted analytical platforms increasingly integrated with them—present unique challenges when it comes to ethical and privacy concerns in conditions like NCSE and SE. EEG signals, unlike other clinical data, are distinctive enough to serve as biometric identifiers. Research shows that these brainwave patterns can be used to identify individuals, even across different datasets or use cases (67). Beyond the EEG waveforms, the files often carry embedded metadata such as timestamps, device IDs, and hospital codes, which can lead to re-identification risks, even if standard de-identification methods are applied (68). Traditional de-identification protocols, usually designed for more structured data, fall short when applied to continuous neurophysiological recordings like EEG, underscoring the need for more specialized methods. In the United States, EEG data does not automatically fall within the scope of HIPAA. Rather, HIPAA applies only where such data qualify as individually identifiable health information held by a covered entity or business associate. In the European Union, EEG and other neural data may, depending on the context and purpose of processing, fall within the GDPR’s special categories of personal data, particularly health data and, in some cases, biometric data. As Yang & Jiang note, however, these existing categories capture neural data only imperfectly and do not fully address the range of scenarios raised by BCI-related data processing (69). Notably, the GDPR prohibits transferring sensitive data to regions without adequate data protection, which can complicate international collaborations. Federated learning has therefore been proposed as a privacy-preserving alternative, allowing institutions to keep data locally while sharing only model updates for aggregation (70).

The integration of AI into these systems also raises concerns about automation bias. Clinicians, especially those with limited experience reading EEGs, may rely too heavily on AI alerts, which can lead to unnecessary treatment escalation or missed diagnoses. CADTH notes that non-specialists may treat AI outputs as definitive, increasing the risk of over- or undertreatment (71). AI should therefore be used as a support tool, with experienced clinicians retaining responsibility for interpreting the findings in context. In emergency and prehospital settings, obtaining informed consent for rapid EEG may be challenging, as patients are often unable to provide valid consent because of impaired consciousness or altered mental status. Despite its non-invasive nature and low procedural risk, rapid EEG involves the collection and handling of neurophysiological data and therefore requires transparent governance and appropriate data-protection measures (72, 73). Importantly, access to AI-enabled EEG tools remains uneven across clinical settings. They are often more available in well-resourced hospitals than in rural or underserved areas. This is particularly important in NCSE, where early detection is critical. Broader implementation will be important to ensure that these technologies reduce, rather than deepen, existing disparities in care (31, 33, 42).

### 5.4 Interdisciplinary and implementation challenges

Implementing rapid EEG—and, where applicable, its AI-assisted analytical components—extends beyond technical feasibility and requires reconfiguration of clinical workflows and coordination across multiple disciplines (12, 20, 74, 75). Along the prehospital-to-emergency continuum, patients may undergo rapid EEG acquisition in the field, receive preliminary AI-supported interpretation, and subsequently transition to emergency departments where neurologists review data remotely and determine the need for cEEG or ICU admission (20, 32, 50, 60). Effective implementation requires alignment across several domains: EMS protocols must define indications for EEG deployment and data transmission; emergency department workflows must accommodate real-time EEG review within triage processes; and neurology or neurocritical care teams must be available to respond promptly to high-risk findings, often via teleconsultation. Any weak link—such as insufficient training, technical failure, or delayed expert review—can disrupt the entire diagnostic chain (12, 32, 50, 61). Additional barriers include personnel training and staffing models, integration of telemedicine and data infrastructure, device maintenance and consumable logistics, and uncertainty surrounding reimbursement and cost-effectiveness (31, 76, 77). Although early economic analyses suggest that rapid EEG may reduce unnecessary ICU admissions, interfacility transfers, and length of stay, robust cost-effectiveness data remain limited and context dependent (78–80). Addressing these challenges requires sustained multidisciplinary collaboration involving neurologists, emergency physicians, intensivists, neurodiagnostic technologists, engineers, data scientists, and healthcare administrators. Continuous monitoring of real-world performance metrics—such as time to EEG acquisition, treatment decision latency, seizure detection rates, avoidance of overtreatment, and patient outcomes—is essential to guide iterative workflow optimization (20, 60, 61, 80).

## 6 Future developments and prospects

As AI technologies mature, the integration of rapid EEG with AI holds promise for improving early recognition and triage of SE and NCSE across the prehospital-to-emergency continuum. A near-term priority is the development of tiered, human–machine collaborative workflows that combine rapid EEG acquisition, AI-supported risk stratification, and timely expert confirmation. A pragmatic pathway may include rapid EEG deployment within minutes of first medical contact, real-time AI outputs categorizing patients into low-, intermediate-, or high-risk groups, and predefined escalation to neurologist review and cEEG for high-risk or uncertain cases (11, 37, 38).

Benzodiazepines remain the mainstay of most prehospital protocols for status epilepticus. When seizures persist despite adequate initial benzodiazepine therapy, timely escalation to a second-line agent becomes clinically important (81, 82). In ESETT, levetiracetam, fosphenytoin, and valproate showed similar efficacy as second-line treatments, with each achieving seizure control in about half of benzodiazepine-refractory cases (82). Among these agents, levetiracetam is often favored in the prehospital setting, because it is easy to dose, can be infused rapidly, has few clinically relevant drug interactions, and does not carry the same infusion-related cardiovascular monitoring burden as fosphenytoin (83–85). Prehospital studies suggest that it can be administered feasibly in the field, although a clear efficacy advantage over benzodiazepines alone has not yet been established (84, 85). In this context, AI-enabled point-of-care EEG may have a role as an adjunct during transport, particularly in patients with persistent impaired consciousness or suspected ongoing electrographic seizure activity, by helping identify those who may warrant earlier escalation to second-line therapy (12, 31). For now, however, this approach remains investigational and should be presented as a strategy requiring prospective validation rather than as an established prehospital standard.

Beyond acute triage, future AI models are likely to incorporate multimodal data—including EEG, neuroimaging, physiological signals, and biomarkers—to better capture the spatial and temporal dynamics of epileptic networks (86–88). In the prehospital and emergency context specifically, combining real-time EEG with point-of-care blood biomarkers, vital sign trends, and structured clinical scores could improve risk stratification accuracy beyond what any single modality achieves alone.

A less discussed but clinically important frontier is the differentiation of epileptic seizures from their mimics in the emergency setting. Psychogenic non-epileptic events (PNES) account for 20–35% of epilepsy monitoring unit diagnoses, and misidentification carries real consequences: a pooled analysis of the RAMPART and ESETT trials found that 8.1% of patients treated for status epilepticus were ultimately diagnosed with PNES, with adverse effects including respiratory depression and intubation documented in 26–33% of cases (89). Rapid EEG could introduce objective electrographic evidence into this decision point—not to diagnose PNES in the field, which still requires video-EEG, but to flag the absence of epileptiform activity before committing to aggressive pharmacotherapy. Machine learning approaches to PNES–epilepsy differentiation based on EEG features have shown early feasibility, though prospective validation in emergency populations is still needed (90).

In parallel, the establishment of a standardized outcome reporting framework is essential. Such a framework should incorporate both process measures, including time to EEG acquisition and time to treatment decision, and patient-centered outcomes, such as mortality, functional independence, quality of life, and long-term seizure freedom. Standardized reporting would facilitate comparisons across studies and support future large-scale meta-analyses. Longitudinal tracking studies aim to evaluate whether early artificial intelligence-based epileptic seizure detection technology can reduce the incidence of epilepsy, cognitive decline, or the recurrence rate of epileptic seizures. This is also a crucial step in shifting epilepsy treatment from passive diagnosis to active prediction and prevention. Future research should prioritize multicenter clinical validation, interpretable model design, ethical governance, and equity considerations to ensure that AI-enabled rapid EEG delivers tangible benefits without unintended harm (31, 44). Through interdisciplinary collaboration and evidence-based validation processes, a continuous identification and intervention loop from prehospital to emergency care can be established, enhancing the clinical application value of AI and rapid EEG technology in epilepsy management (2, 10, 15, 91). The optimal paradigm is not AI replacing experts, but a human–machine partnership that supports timely, evidence-based decisions across the entire acute care pathway.
